# Overview of Bone Marrow Aspiration from 120 Cats in Different Hematological Conditions

**DOI:** 10.1155/2023/2493618

**Published:** 2023-08-22

**Authors:** Thierry G. de Cristo, Giovana Biezus, Geanice Ledo, Marcela B. S. Teixeira, Mayara Vavassori, Denilson R. Soares, Mere E. Saito, Renata A. Casagrande

**Affiliations:** ^1^Laboratório de Patologia Animal (LAPA), Universidade do Estado de Santa Catarina (UDESC), Lages, SC, Brazil; ^2^Centro de Controle de Zoonoses, Lages, SC, Brazil; ^3^Laboratório de Patologia Clínica Veterinária (LPCV), Hospital de Clínicas Veterinárias (HCV), UDESC, Lages, SC, Brazil

## Abstract

Bone marrow (BM) evaluation is highly important for the diagnosis of numerous hematological alterations in animals, especially cats, given their greater propensity for hematopoietic changes associated with retrovirus infections. This study aims to describe the main aspects of the BM of cats with different hematological conditions, comparing with reference intervals established from animals without hematological alterations and also with a previously established reference interval. To do so, we evaluated full blood and BM samples from 120 cats with no abnormalities on physical examination, negative for FeLV and FIV. Hemograms were performed from automated total cell and platelet and leukocyte differential counts in blood smears. BM samples were placed in Petri dishes; medullary spicules were selected to make up to eight cytological slides stained by the May–Grunwald–Giemsa technique, which were subjected to quantitative and cellular morphology evaluations. The cats were predominantly young, 64.2% female and 35.8% male. The average number of medullary spicules in samples was 13.7%, and density was 44%. In the BM quantitative analysis, prorubricytes and rubriblasts had higher quantities than the recommended one for all cats with or without hematological alterations. In all groups, lymphocytes were decreased, and cats with lymphocytosis were closest to the reference range, showing flame cells and Mott cells. The reference interval for the bone marrow cell count obtained from the samples in this work differs from previously established data, leading to different interpretations of the patient's BM condition, according to the cell population observed and the reference interval used. This divergence strongly emphasizes the need to correlate clinical, epidemiological, hematological, and bone marrow data of each patient for a better understanding of the patient's condition. The popularization of BM assessment is essential so that more reliable reference intervals can be established according to the population served by each pathologist and clinical laboratory.

## 1. Introduction

Bone marrow evaluation is a valuable resource to identify diseases associated with the production, maturation, and release of erythrocytes, leukocytes, and megakaryocytes (MKs). It allows us to identify changes in such cells directly at their site of origin [1, 2]. In veterinary medicine, despite being a diagnostic tool not often listed by the clinical community, BM aspiration is more commonly used than biopsy due to its faster analytical speed and ease of sampling [1, 3].

Bone marrow examination should be performed in conjunction with hematological evaluation and consists of two main and complementary analyses, morphological and quantitative [2–4]. The former seeks to characterize cells in terms of morphology, identifying cytoplasmic and nuclear alterations, while the latter seeks a broad view of medullary composition, indicating numerical variation of precursor cells and their subsequent lineages [2].

In cats, BM evaluation is recommended in cases of persistent regenerative anemia, neutropenia, and thrombocytopenia, as well as undefined leukocytosis, morphological abnormalities in circulating leukocytes, erythrocytes, and platelets [5–11]. It is also of great diagnostic value in cases of suspicions of BM dysfunctions due to primary or metastatic neoplasms, or associated with feline leukemia virus (FeLV) and feline immunodeficiency virus (FIV) infections [8–11].

Despite the importance of BM evaluation in this species, reference intervals commonly used date from the 1960s and 1990s and considered results from a small number of animals [2, 5, 12]. In addition, there is a lack of work on cat BM quantitatively, usually focused on specific changes such as parasitic and viral neoplastic infections [6, 7, 13–15].

This work aims to describe the cytological aspects and differential counts of BM samples obtained in a short time from a population of randomly selected cats in the mountain region of Santa Catarina State (SC), in Brazil, according to hematological abnormalities expressed by them.

## 2. Materials and Methods

Blood and BM samples were collected from 188 cats selected randomly from routine care and routine neutering performed at the Zoonosis Control Center (ZCC) of the Municipal Health Secretariat and clinical care at the Veterinary Clinics Hospital (VCH) of the State University of Santa Catarina State (UDESC, in Portuguese), both in the city of Lages, SC, Brazil. The sampling was made between April and December 2019. These patients came from a community care routine for animals from low-income families; therefore, they were not evaluated before the day of the procedure. There was no restriction on the sex, age, or breed of the evaluated patients [16].

The animal age range was determined according to the life stage guidelines for feline species of the American Association of Feline Practitioners (AAFP) and American Animal Hospital Association (AAHA) [17]. Accordingly, the cats were classified as a kitten (0–6 months), young (7 months–2 years), young adult (3–6 years), adult (7–10 years), senior adult (11–14 years), and elderly (over 15 years).

Clinically debilitated cats, with macroscopically expressed diseases or histories of recent diseases evidenced from the physical examination, were excluded from the study, as well as animals in which *Mycoplasma* spp. infection was suspected during the evaluation of blood smears, and those positive for FeLV or FIV.

Testing for FeLV and FIV was performed using the enzyme-linked immunosorbent assay (ELISA; SNAP™ FIV/FeLV Combo Test, IDEXX Laboratories, Inc., Westbrook, Maine, USA) and additionally for FeLV by polymerase chain reaction (PCR). FeLV proviral DNA was amplified based on the U3 LTR region and gag gene of FeLV subgroup A. Of the 188 cats evaluated, 49 were positive for FeLV and two mutually positive for FeLV and FIV.

Blood for a complete blood count (CBC) was collected by jugular venipuncture, and samples were stored in tubes with EDTA for a maximum of 5 hours in refrigeration (ranging from 4 to 8°C) until the moment of analysis. Hematocrit was determined by the microhematocrit method. A total count of erythrocytes and leukocytes was performed using an automated cell counter (SDH-3 Vet, Labtest, Lagoa Santa, Minas Gerais, Brazil). The differential leukocyte count and estimated platelets were counted from stained blood smears with rapid hematological stains (Newprov, Pinhais, Paraná, Brazil). Evaluation and interpretation of changes in the CBC were performed using reference intervals (RIs) established by Weiss and Wardrop [8] since these are the RI values routinely used in the sampled region, and no specific criteria were used to establish an interval for hematology from the animals evaluated in this research.

For BM sampling, the cats were maintained under general dissociative anesthesia with ketamine and xylazine by intramuscular (IM) injection at 10 mg/kg and 1.5 mg/kg, respectively. Tramadol (2 mg/kg/IM) was used as a preanesthetic medication, and preventive antibiotic therapy was made with benzathine penicillin (24 mg/kg/IM). After the sampling, the patients rested until complete anesthetic recovery, followed by surgical discharge. The patients were followed up by telephone contact with the tutors and returned after seven days for removal of the sutures of the neutering procedure and general clinical evaluation. None of these patients showed any relevant alterations from the immediate postoperative period until the removal of the stitches.

Bone marrow samples were collected from the left deltoid tuberosity using standard Jamshidi trifaceted or Illinois beveled cannulas, with a Luer-Lock closure system and size of 16G (0BIL1617/080™ and BIL1617™, Biomedical Srl, Firenze, Italy). Aspiration was performed using 5–10 mL syringes coupled to the cannula, to obtain 0.3–0.8 mL of BM. At least five and at most eight marrow smears were prepared per patient using the squash technique, depending on the number of spicules obtained. All smears were stained using the May–Grunwald–Giemsa (MGG) technique diluted in Sorensen buffer. The smears were first evaluated according to the number of spicules per smear (×100), density and cellularity of spicules (number of precursors/adipocytes), degree of maturation of precursors, and quantity of lymphocytes and plasma cells in every five fields of higher magnification (×400).

A cytomorphological evaluation was performed according to Harvey [1]. Megakaryocytes were carefully evaluated at 100x magnification in 10 fields of the perispicular and spicular region. Quantitative differential evaluation of BM was performed on the two best smears of each animal, which were free of artifacts and without excessive cellular overlap, by counting 500 nucleated cells (NCCs) at 1000x magnification in immersion, with the aid of a light optical microscope. The counts were cataloged and calculated using the BM conter©2008 smartphone application (Kazuyoshi Sasaoka, AppStore).

The differential cell count was performed in hot spots on the periphery of spicules, having reference values as previously described by Jain [5] and the NCC of the cats evaluated in this research free of clinical and hematological alterations. Among all the collected samples, 17 had NCC lower than 500 or showed marked platelet aggregation and, therefore, were excluded from the analysis. Cells were carefully evaluated for size, maturation degree, cytoplasmic color, nuclear shape, nuclear segmentation, chromatin arrangement, and cytoplasmic and cytoplasmic membrane characteristics, with any changes being described as prescribed by Harvey [1].

The bone marrows were considered regular if they showed cellularity ranging between 25% and 75% concerning the number of precursors/adipocytes, widely dependent on the age of the animal in question, in which the myeloid to erythroid ratio (MER) decreases proportionally with advancing age. These results were always analyzed in conjunction with the CBC of the respective patient.

Due to the limitations of this research, the promoting factors of the patient's hematological alterations were not investigated. Thus, the groups for comparison were composed solely according to the alteration observed, without focusing specifically on the cause. For comparison with BM obtained data, the evaluated animal groups were selected and separated according to hematological condition in cats without hematological abnormalities, anemic, with neutrophilia, eosinophilia and basophilia, lymphocytosis, monocytosis, and lymphopenia, considering that the same animal could be part of more than one group.

The obtained data were compiled in Excel™ spreadsheets (2019) for descriptive and inferential statistical analysis, determining means, standard deviations, and percentiles (25%–75%), using Jamovi® statistical software version 2.0 (Jamovi Project Computer Software, Sydney, Australia).

The samples of BM, blood, and epidemiological data used in this manuscript came from a sample from another research project carried out by the same authors, which was approved by the Committee on Ethics in the Use of Animals (CEUA) of the University of Santa Catarina State (UDESC), with registration 2306130319 (ID 000831). In addition, an informed consent form was signed by the owners authorizing the use of the samples and data provided.

## 3. Results

Among the 120 cats, 64.2% (77/120) were females and 35.8% (43/120) were males. As for age groups, 17.5% (21/120) were kittens, 67.5% (81/120) were young, 12.5% (15/120) were young adults, and 2.5% (3/120) were adults. There were no animals in the other age groups. Furthermore, none of the animals from the VCH routine could be included in this study, as they had clinical diseases.

When evaluating CBC, 47% (57/120) of patients showed no hematological abnormalities, but alterations were observed in the remaining 53% (63/120) patients. Notably, more than one alteration could be found in each patient such as eosinophilia (23/120; 19.2%), neutrophilia (22/120; 18.3%), thrombocytopenia (20/120; 16.7%), basophilia (15/120; 12.5%), monocytosis (12/120; 10%), lymphocytosis (8/120; 6.7%), anemia (2/120; 1.7%), and leukopenia (2/120; 1.7%).

As for the overall analysis of 120 BM aspirates, the number of spicules per sample ranged from 1 to 50 per slide, with an average of 13.7 (±8.7), and density between 25% and 80%, with an average of 44% (±15.3). The average number of spicules in young animals was 8.1 (±5.1), with an average density of 42.1% (±14.1); in adult animals, the number of spicules reached an average of 16.3 (±7.2) and an average density of 56.7% (±50). The count of MK showed an average of 16.9 (±8.8) cells per ten low-power fields (10x objective) and that of lymphocytes had an average of 5.31 (±1.8). In rare cases, MKs with neutrophil emperipolesis were observed.

None of the animals submitted to this study presented any illness at the time of sampling or afterward. During the telephone follow-up carried out with the tutors, none of the patients had changes in their health status or behavior after the procedure, until the removal of the suture stitches.

### 3.1. Cats without Hematological Changes

Among the 57 (47%) patients without hematological changes, 68.4% (39/57) were females and 31.6% (18/57) were males, with ages ranging from 5 to 108 months ($\bar{X}$ 18.8 ± 19.6), among which the young animals predominated (56.1%; 32/57), followed by kittens (26.3%; 15/57), young adults (15.8%; 9/57), and adults (1.8%; 1/57).  Table 1 shows the CBC findings of the patients.

By evaluating the BM myeloid series, the numbers of myeloblasts ($\bar{\times }$ 1.6% ± 0.8), promyelocytes ($\bar{\times }$ 3.6% ± 1.7), and neutrophils ($\bar{\times }$ 26.3% ± 4.1) were superior to those previously described. On the other hand, in the erythroid lineage, the rubriblast count ($\bar{\times }$ 1.1% ± 0.5) and the prorubricyte count ($\bar{\times }$ 4.9% ± 2) were higher than the IR used. The averages of lymphocytes and plasma cells observed in the sample were 9% (±3) and 1.4% (±1.3), respectively. The MER in patients ranged from 0.9 to 2.2, with an average of 1.49.  Table 2 describes the data on the analysis of the BM of cats.

### 3.2. Cats with Hematological Changes

This group comprises the animals that presented some hematological alterations, even in the absence of clinical signs or a history of previous illnesses. None of these patients developed a clinical disease until the end of the postsurgical period, nor did the tutors seek clinical care or report any abnormal health conditions in the patients until the completion of this project.

### 3.3. Anemia

The two cats that had changes compatible with anemia were males, young, aged 24 months old. The cause of anemia was not investigated, as these patients had no history or evident clinical alterations at the time of obtaining the samples, one of them with normochromic normocytic anemia (Cat 1) and the other with normochromic microcytic anemia (Cat 2), as shown in Table 3, both without relevant leukocyte alterations, in addition to mild eosinophilia. Neither of the two samples underwent reticulocyte counts at the time of CBC.

**Figure 1:**
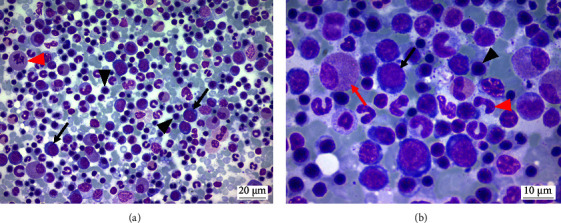
Bone marrow aspirate from a cat with erythroid hyperplasia. (a) Rubriblasts (black arrows) in moderate quantity, followed by a large number of rubricytes (black arrowheads) at different maturation stages, and a large granulocytic precursor in typical metaphase (red arrowhead) (400x, MGG). (b) Approximate image A in which increasing proportions of rubriblasts (black arrow), prorubricytes (black arrowhead), and multistage rubricytes can be seen regarding granulocytic precursors, such as myelocytes (red arrow) and rod neutrophils (arrowhead red) (1000x, MGG).

In both patients, the erythroid lineage was superior to the myeloid, with a special prevalence of prorubricytes (11.6% and 9.8%), about ten times higher than that recommended by Jain [5] and slightly increased about to the values obtained in the hematologically healthy animals of this research (Table 4). Both patients had MER below 1 (0.95 and 0.88, respectively), contrasting with the values indicated in the recommended and self-instituted references. The erythroid lineage showed reactive hyperplasia in both cases (Figures 1(a) and 1(b)).

### 3.4. Neutrophilia

Neutrophilia was observed in 18.3% (22/120) of cats. Of these, 63.6% (14/22) were males and 36.4% (6/22) were females, with an average age of 20.3 (±13.8) months and predominance of young age animals (81.8%; 18/22). The average number of neutrophils observed was 17.506/*µ*l (±3.375), with no evidence of rod or hypersegmented ones.

Isolated neutrophilia was observed in eight (36.7%) cases, the others were associated with eosinophilia and monocytosis (31.8%; 7/22), eosinophilia (13.6%; 3/22), lymphocytosis (9.1%; 2/22), and lymphocytosis with eosinophilia, lymphocytosis with monocytosis, or isolated monocytosis with 4.5% (1/22) each. Of the 22 animals, 21 had numbers of intramedullary neutrophils above the maximum indicated for the reference interval, which is compatible with a picture of granulocytic hyperplasia of the neutrophilic order. However, using the values obtained in the 57 cats free of hematological alterations as a reference, the number of intramedullary neutrophils is found in values considered within a normality threshold, despite the average value of rods being slightly increased. A general marrow analysis of cats with neutrophilia can be seen in Table 5.

### 3.5. Eosinophilia and Basophilia

Increased numbers of circulating eosinophils were identified in 19.2% (23/120) of cats. Of these, 78.3% (18/23) were females and 21.7% (5/23) were males. The average age of these animals was 20.6 (±13.9) months, among which young cats accounted for 73.9% (17/23) of cases. These cats had no changes in the erythrogram; however, 12 (52%) had leukocytosis (19.960 to 35.550/*µ*l). The average amount of eosinophils observed in these patients was 2.827/*µ*l (±1.692). Quantitative analysis of the BM in these cats was very similar to that in neutrophilia patients, except for prorubricytes ($\bar{\times }$ 6.8% ± 2.9) and the sum of the average number of eosinophilic lineage cells (∑ $\bar{\times }$ 3.8%), both increased when compared to the previously reported RI. In the data obtained from the hematologically healthy patients, the eosinophil precursors were separated according to each specific cell type, and when comparing patients with eosinophilia with these data, no relevant quantitative changes were observed.

Among cats with eosinophilia, there were still two previously mentioned cases of anemic animals with erythroid hyperplasia (8.7%, 2/23) and 21 (91.3%) cases of granulocytic hyperplasia of the neutrophilic order. Among these 21, eight (38.1%) also had several intramedullary eosinophils (summing up all maturation phases) higher than the recommended reference interval determined by Jain [5], but not concerning the reference established in this research, as can be seen in Table 6.

Regarding basophils, only 12.5% (15/120) of cats showed basophilia, of which females totaled 66.7% (10/15) and males were 33.3% (5/15), with an average age of 15.4 (±1.3) months. The young age group was predominant (80%; 12/15), followed by kittens (13.3%; 2/15) and young adults (6.7%; 1/15). These patients had an average of 407/*µ*l circulating basophils (±321), ranging from 101 to 1.259/*µ*l.

Quantitative analysis of the BM in these patients was similar to that previously mentioned, with a clear increase in the number of erythroid lineage cells, if the recommended reference was used, however, without changes when using the one established by the animals without hematological alterations in this research. Despite basophilia, there was no increase in the average of basophil precursors ($\bar{\times }$ 0.3% ± 0.3) compared to animals with no one or other hematological conditions. The analysis also showed that, among these cases, 14 (93.3%) had neutrophilic granulocytic hyperplasia as recommended by Jain [5], in addition to those with erythroid hyperplasia.

### 3.6. Lymphocytosis

Eight (6.7%) animals presented lymphocytosis, divided equally between males and females, of which 87.5% (7/8) were young. Lymphocytes in these patients ranged from 7.096 to 12.658/*µ*l ($\bar{\times }$ 8.735/*µ*l ±1.739). None of the animals had isolated lymphocytosis.

Compared with the previously established reference intervals, the evaluation of the bone marrow of these animals showed increasing numbers of myeloblasts ($\bar{\times }$ 1.5% ± 0.7), promyelocytes ($\bar{\times }$ 3.1% ± 1.6), and neutrophils ($\bar{\times }$ 25.9% ± 2.4). Conversely, for erythroid lineage cells, only prorubricytes had higher numbers, averaging 5.7% (±2). Lymphocytes were also not abundant in BM smears, but intramedullary lymphocytes had a number closer to that recommended by Jain [5], with an average of 10.2% (±4). The reference instituted from the use of data from cats in this research did not demonstrate relevant quantitative alterations in any of the aforementioned cells. The general appearance of the bone marrow of these animals is shown in Table 7.

**Figure 2:**
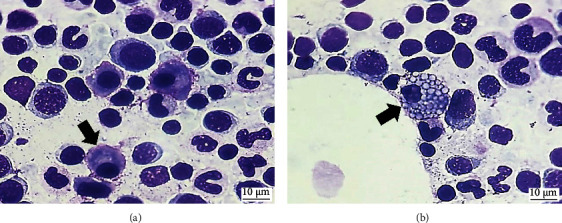
Bone marrow aspirates from cats with lymphocytosis. (a) Abundant plasma cells with the irregular cytoplasmic membrane and cytoplasm with intensely pinkish margins, characterizing flame cells (black arrow). (b) Large plasma cells filled with optically empty vesicles of similar size (Russell's corpuscles), characterizing a Mott cell (black arrow) (1000x, MGG).

Moreover, four of these cats (50%, 4/8) had discreetly increased plasma cells in the marrow, ranging from 1.6 to 2.4% ($\bar{\times }$ 2% ± 0.4), according to Jain [5], but not with the values established in the research. Flame cells could also be identified in these cases (Figure 2(a)) and Mott cells in two cases (25%) (Figure 2(b)).

### 3.7. Monocytosis

Monocytes increased in 12 (10%) cats, among which females were more (7/12; 58.3%), with an average age of 20.8 (±13.8) months. Again, young animals predominated, with 66.7% (8/12) of cases. Monocytosis ranged from 1.048 to 2.028/*µ*l, with an average of 1.361/*µ*l (±1.269/*µ*l). Although no changes were observed in the erythrogram, the average number of platelets observed was 301 × 10³/*µ*l, which is quite close to the minimum platelet threshold [8].

Leukocytosis was detected in 10 (83.3%) of these animals, in which, in addition to monocytosis, neutrophilia predominated (9/12; 75%), followed by eosinophilia (8/12; 66.8%), basophilia (6/12; 50%), and lymphocytosis (2/12; 16.8%). Cytological evaluation of the BM revealed monocyte values higher than the maximum limit of the reference used (0.0–0.2), ranging from 0.4 to 2.2%, with an average of 1.6% (±1.1%). Only one patient had a monocyte percentage of 2.2%, higher than the reference interval established by the authors of this manuscript. In one case, a large number of neutrophils were recorded. The differential count of the evaluated cells is better described in Table 8.

### 3.8. Lymphopenia

Lymphopenia was observed in 17 (14.2%) animals, 64.7% (11/17) of which were females and 35.3% (6/17) males, with an average age of 27.4 (±29.1) months, ranging from 6 to 108 months. Their lymphocyte values ranged from 480/*µ*l to 1,493/*µ*l, which is close to the lower minimum limit, with an average of 1,129/*µ*l (±279/*µ*l). Neutrophilia was noted in four (23.5%) of these 17 animals, not exceeding 14,370 neutrophils/*µ*l.

The average total lymphocyte count in BM aspirates was 8.2% (±3.1), ranging from 1.2% to 13.6%. Among medullary smears from these cats, only two (11.76%) had lymphocyte values within the expected by Jain [5], all the others showed lower values, and furthermore, some of these animals also had concomitant monocytosis. These data contrast with the interval values obtained by the authors, which range from 6% to 12% and cover 13 of the lymphopenic animals. No morphological anomalies were observed in lymphoid, myeloid, or erythroid precursors, in any of the 17 marrow samples. The general appearance of the BM of these cats is shown in Table 9.

## 4. Discussion

As for the general evaluation of aspirates, our findings showed that the number of medullary spicules was higher in adult animals, contradicting what was expected when considering the physiological characteristics of patients [1]. Younger cats are known to have greater medullary activity and availability of spicules for evaluation [1, 18]. However, in this study, kittens showed a smaller number of spicules in the sample than other age groups. It is also important to point out that factors such as the technique and sample obtained may also influence this result. Artifacts in the manufacture of slides can compromise the distribution of cells, culminating in intense overlapping, crushing, and countless others, significantly interfering in the analysis [19].

Samples of males had a higher number of viable spicules than those of females. A study in humans evaluating BM aspirated nucleated cells identified a progressive reduction in their number with advancing age; however, no significant differences were reported between the sexes [20]. So far, no studies have discussed variations in the number of spicules between sexes and age groups in cats. For us, the relationship between these factors and counts of spicules in samples is unlikely, thus occasional finding.

In adult cats, MKs were numerically superior to those in other age groups. This finding was also reported in a study on megakaryocytic activity in mice; the authors believed that it is related to an increase in thrombopoietin activity and its respective receptor [21]. In rare cases, emperipolesis of neutrophils could be observed in mature megakaryocytes, as already described in humans [22]. The process is still poorly understood but is believed to be linked to the limited space restriction in the BM, or even the access to the circulation route [23–25]. In addition, MK emperipolesis can be observed in myeloproliferative diseases, involving distinct leukemia [26, 27]. However, those alterations were not diagnosed in the animals in our study.

The 57 cats without hematological changes had total marrow cell counts different from those described by Jain [5]. They also differed from those found by Turinelli and Gavazza [11], in a retrospective study where the most evident discrepancy was for total values of myeloid and erythroid precursors.

In our study, in turn, these were about 10% higher for myeloid lineage and 10% lower for the erythroid one. Although physically healthy, most of the animals evaluated in this study came from routine neutering made in ZCC-Lages. This surveillance center serves, for the most part, a socially vulnerable population; thus, control and prophylaxis measures against animal diseases are uncommon. Because of this, such constant and intense exposure to immunological challenges is speculated to promote continuous stimulus to granulopoiesis [1, 2, 9]. As the sampling was carried out in a single moment, subsequent samples were not collected for comparison purposes, limiting the evaluation of the evolution of the hematological condition of these patients.

Environment-related hematopoietic changes have also been described in sheep exposed to high temperatures [28]. Still, no study has assessed the influence of environmental factors on cats. Therefore, granulopoiesis observed in our study seems to be an adaptive response to the environment where these animals lived, increasing numbers of intramedullary myeloid precursors.

The MER in animals without hematological changes ranged from 0.9 to 2.24, with the minimum being lower than the recommended one (1.21) [5]. This result may lead the evaluator to consider a condition compatible with erythroid hyperplasia or granulocytic hypoplasia. Thus, MER must be judiciously evaluated and in association with other medullary, hematological, and clinical alterations to determine the existence of a disease associated with the condition or if these results are inherent to the patients themselves [29, 30]. Other studies on normal cellularity in the BM of cats reported MER values even lower than those found in this work, ranging from 0.31 to 0.6 [11, 12]. It is also noteworthy that the reference interval reported by Jain [5] is based on a small number of animals from a specific region.

Another important observation is related to the average number of eosinophilic precursors in these cats, which together exceeded the maximum stipulated by Jain [5] by about 25%. Studies have revealed that hyperplasia of this lineage can only be determined when precursor values exceed 6% of the neutrophil count [1, 2, 9, 11]. This finding, therefore, was not considered relevant when evaluated in conjunction with the blood count. Furthermore, these precursors were typical and without significant morphological, nuclear, or cytoplasmic alterations that are indicative of dysgranulopoiesis [4, 9]. It is also not possible to rule out that such an alteration is simply physiological and transitory.

Both anemic patients exhibited reactive erythroid hyperplasia, indicating a marrow response. This feature requires at least four days to occur, a period required for erythropoietin action to be efficient. It is said that such conditions can be determined by low MER values, in association with a large number of nucleated erythrocytic cells in aspirates, among which rubricytes and metarubricytes predominate [9]. It differs from what was observed by the authors, in which rubriblasts had larger amounts. Such a hyperplastic response, associated with anemia, may represent a subclinical chronic disease, which can range from iron deficiency to progressive renal diseases [5, 8, 31]. Nevertheless, a more assertive diagnosis was limited by the method used in this research.

Neutrophilic cats, in turn, showed MER within the reference range, but the average of intramedullary neutrophils was above the standard. Therefore, these cats could be characterized as carriers of neutrophilic granulocytic hyperplasia [5, 9]. Such an alteration may require more days to significantly alter MER [9], which could explain why values remained within the prescribed range and the hematological healthy animals in this research. However, the absence of clinical and hematological features of inflammatory and infectious processes, in most of the evaluated cats, makes us question whether granulocyte values prescribed for hyperplasia diagnosis are overestimated.

The number of intramedullary or circulating neutrophil precursors showed no changes. In this way, such hematological findings can be associated with the conditions in which animals remained from transport to sample collection. They were far from their natural environment, sharing space with people and animals different from usual, and being subjected to climate changes, mechanical restraint, and manipulation [32–34].

Eosinophilia and basophilia were observed in multiple patients. However, they cannot be attributed solely to physiological leukocytosis, as the parasite load and history of allergic diseases or hypereosinophilic syndromes were unknown by their owners. Studies have described those helminths, autoimmune syndromes, and allergic diseases are the main promoters of these hematological changes. Moreover, stimulation of granulocytic colonies of eosinophils and basophils (GC-Eo/B) by interleukin 5 (IL-5) action may or may not increase eosinophilic cellularity [5, 33–37].

Few studies numerically exposing normal cat marrow have pointed to a wide variation in intramedullary eosinophilic precursors but with an average of no more than 3.2% of NCC [5, 11, 12]. Interestingly, cats without hematological alterations had a higher number of intramedullary eosinophilic precursors. This result led us to question whether the reference range of these cells for cats is not strongly dependent on the living conditions and habitat of the patients, as previously discussed. This is because it induces a physiological need for a greater supply of eosinophils. This hypothesis is based on data from another study, which showed a maximum of 7% for eosinophilic lineage cells in animals with BM under normal conditions [11]. It contradicts the values previously defined to consider eosinophilic hyperplasia [7, 9].

The marrow response in cases of lymphocytosis was also not expressive in any of the cats evaluated in this study. Of the eight animals with such an alteration, four showed a simultaneous increase in neutrophils, in line with what is expected in the case of lymphocytosis due to excitation [32–34]. Although lymphocytosis is often a nonspecific finding, it may also be associated with viral infections, hemoparasites, and mild inflammation, or with certain chronicity [33, 34] and even chronic lymphocytic leukemia (CLL) [38, 39].

Under conditions described as normal, marrow lymphocytes can vary from five to 21.6% of NCC [2, 5, 30], which is in line with the data found in this research. These values seem to be high when compared with older data, which estimated an average of 5% of lymphocytes in the BM [12], as well as in the reference instituted by the authors in the elaboration of this research; however, they are close to the average value found by other authors [13]. According to the literature, mature lymphocytes are easily confused with erythroid precursors due to their size and shape [2, 29, 30]. However, we emphasize the need to pay attention to cytoplasmic features and erythroid cell nuclei, which tend to be intensely basophilic and with more compacted chromatin, as described by other authors [4].

Mott and flame cells, which are deemed as reactive forms of plasma cells, were observed in cats with lymphocytosis in moderate amounts, with the flame cells being the most numerous. Flame cells consist of larger than usual plasma cells, with reddish inner cytoplasmic membranes due to the accumulation of carbohydrate-rich immunoglobulins, predominantly IgA [2, 39]. Both flame and Mott cells have been observed as primary components of leukemia in humans [40], as well as in monoclonal gammopathies [41] and myelomas in dogs and cats [42–44]. These patients showed no clinical changes that could indicate the existence of the mentioned diseases. However, due to a limitation of this study, complementary tests with greater specificity were not performed to confirm or exclude those diagnoses, such as the detection of Bence–Jones protein in urine, as well as imaging and serum biochemical evaluations [45, 46].

The same is discussed for the 17 cats showing isolated lymphopenia, without reduction in other circulating cells. There are numerous causes for this, among them viral diseases, rupture of lymphatic ducts, neoplastic processes that alter the structure of lymphatic organs, and use of immunosuppressive drugs [33, 34, 47, 48]. However, none of the cats showed clinical or historical alterations that could support these causes. Furthermore, it is relevant to mention that 13 of these patients had lymphocyte values similar to the reference interval established by the authors, which makes the idea that the number of previously established lymphocytes does not globally represent patients of the feline species feasible. An exception may be viral infections since; in this work, animals were only tested for FeLV and FIV.

Such patients had a lower number of intramedullary lymphocytes than the recommended reference [5]. However, besides being within the values established as a reference by the authors, some researchers have characterized marrows as normal when the number of lymphocytes ranges from 1.5% to 28.5% [11], while others have considered a range from 5% to 20% [29].

## 5. Conclusions

The reference interval for the BM cell count obtained from patients without hematological alterations evaluated in this study differs from previously established data, which may be associated with several intrinsic factors, from individual characteristics to the home environment and life habits.

This divergence leads to multiple different interpretations of the condition of the patient's BM, according to the cellularity present in the sample and the reference interval used. This strongly emphasizes the need to correlate the clinical, epidemiological, hematological, and BM data of each patient for a better diagnostic definition.

Although this work does not intend to determine new reference intervals from the evaluation of animals without hematological alterations, it is believed that it can serve as a complement in the study of the BM of some specific patients. In addition, it is observed that BM evaluation should be more popularized, familiarizing clinicians with sampling methods and benefits brought to patients with hematological disorders. From this, it is believed that more reliable references can be established according to the population assisted by each pathologist and clinical laboratory.

## Figures and Tables

**Table 1:** Complete blood count values of 57 cats with no hematological alterations submitted to bone marrow aspiration.

Parameters	Unity	Mean	S.D.^1^	Median	25%	75%	Reference^*∗*^
Erythrocytes	10^6^/*µ*l	7.7	1.1	7.6	6.9	8.5	5.5∼10
Hemoglobin	g/dL	11	1.5	10.9	9.8	12.1	8∼15
Hematocrit	%	33.7	4.7	32	30	37	24∼45
MCV^2^	fL	44.2	4.5	43.5	41	46.3	39∼55
MCHC³	%	32.5	2.1	32.5	31.2	33	31∼35
Platelets	10^3^/*µ*l	425	104	414	363	486	300∼800

Leukocytes	/*µ*l	10.738	3.265	11.277	7.568	12.890	5.000∼19.500
Rod neutrophils	/*µ*l	0	0	0	0	0	0∼300
Neutrophils	/*µ*l	6.502	2.671	6.732	4.069	8.154	2.500∼12.500
Lymphocytes	/*µ*l	3.183	1.475	2.884	2.229	3.765	1.500∼7.000
Eosinophils	/*µ*l	768	451	647	494	901	0∼1.500
Basophils	/*µ*l	96.4	202	0	0	124	Rare
Monocytes	/*µ*l	189	186	124	75	289	0∼850

^1^Standard deviation. ^2^Mean cell volume. ³Mean corpuscular hemoglobin concentration. ^*∗*^Weiss and Wardrop [8].

**Table 2:** Descriptive analysis of the quantitative evaluation of bone marrow aspirates from 57 cats without alterations in complete blood counts.

Lineage	Evaluated cells (%)	Mean	S.D.^1^	Median	25%	75%	pRI^*∗*^
Myeloid		52.1	4.7	52.6	49	54.4	
	Myeloblast	1.6	0.8	1.6	1	2	0∼0.4
	Promyelocyte	3.6	1.7	3.4	2.6	4.4	0∼3
	Neutrophilic myelocyte	4.5	1.9	4.4	1	6	4.4∼13.2
	Neutrophilic metamyelocyte	3.9	1.4	3.8	3	5	0.6∼8.0
	Rod neutrophil	7.8	2.7	7.6	6	9.4	12.8∼16.6
	Neutrophil	26.3	4.1	25.8	24.2	28.4	6.8∼22.0
	Eosinophilic myelocyte	0.5	0.5	0.4	0.2	0.8	0.8∼3.2 (sum of eosinophilic series)
	Eosinophilic metamyelocyte	0.6	0.6	0.4	0.2	0.8	
	Rod eosinophil	1	0.6	1	0.4	1.4	
	Eosinophil	2	1.2	1.8	1.8	2.6	
	Basophilic series	0.4	0.4	0.4	0	0.4	0∼0.4

Erythroid		35.6	4	35	33.2	37.4	
	Rubriblast	1.1	0.5	1	3.2	5.9	0∼0.8
	Prorubricyte	4.9	2	4.6	3.4	6.4	0∼1.6
	Rubricyte	21.4	3.7	20.2	18.8	23.6	10.2∼29.4
	Metarubricyte	8.4	2.5	8.2	6.8	9.6	1.0∼10.4

Others		12.2	3.7	12	10.8	15	
	Lymphocyte	9	3	8.8	7.6	11	11.6∼21.6
	Plasma cell	1.4	1.3	1	0.4	1.8	0.2∼1.6
	Monocyte	1.4	0.7	1.4	1	2	0∼0.2
	Megakaryocytic series	0.4	0.4	0.4	0.2	0.6	Rare

Myeloid/erythroid ratio		1.49	0.25	1.34	1.54	1.65	1.21∼2.16

^1^Standard deviation. ^*∗*^Reference interval (RI) previously published by Jain [5].

**Table 3:** Red cell count values of 2 cats with anemia submitted to bone marrow aspiration.

Parameters	Unity	Cat 1	Cat 2	Reference^*∗*^
Erythrocytes	10^6^/*µ*l	5.4	5.16	5.5∼10
Hemoglobin	g/dL	8.4	7.2	8∼15
Hematocrit	%	23	20	24∼45
MCV^1^	fL	41.9	37.2	39∼55
MCHC^2^	%	36.5	33	31∼35
Platelets	10^3^/*µ*l	310	420	300∼800

^1^Mean cell volume. ^2^Mean corpuscular hemoglobin concentration. ^*∗*^Weiss and Wardrop [8].

**Table 4:** Quantitative results of cytology evaluation of bone marrow aspirates from two clinical health cats with anemia.

Lineage	Evaluated cells (%)	Cat 1	Cat 2	pIR^*∗*^	eIR^*∗∗*^
Myeloid		44	39.8		
	Myeloblast	2	0.6	0∼0.4	0.8∼2.4
	Promyelocyte	2.4	3	0∼3	1.9∼5.3
	Neutrophilic myelocyte	9.6	2.4	4.4∼13.2	2.6∼6.4
	Neutrophilic metamyelocyte	3	3	0.6∼8.0	2.5∼5.3
	Rod neutrophil	5	7.8	12.8∼16.6	5.1∼10.5
	Neutrophil	19.4	21.4	6.8∼22.0	22.2∼30.4
	Eosinophilic myelocyte	0	0.4	0.8∼3.2 (sum of eosinophilic series)	0∼1
	Eosinophilic metamyelocyte	0	0		0∼1.2
	Rod eosinophil	0.4	0.2		0.4∼1.6
	Eosinophil	1.2	0.8		0.8∼3.2
	Basophilic series	0	0.2	0∼0.4	0∼0.8

Erythroid		46.2	45.2		
	Rubriblast	1.4	1.4	0∼0.8	0.6∼1.6
	Prorubricyte	11.6	9.8	0∼1.6	2.9∼6.9
	Rubricyte	26	25.2	10.2∼29.4	17.7∼25.1
	Metarubricyte	7.2	8.8	1.0∼10.4	5.9∼10.9

Others		9.8	15		
	Lymphocyte	4.4	11.8	11.6∼21.6	6∼12
	Plasma cell	3	1.2	0.2∼1.6	0.1∼2.7
	Monocyte	2	2	0∼0.2	0.7∼2.1
	Megakaryocytic series	0.4	0	Rare	0∼0.8

Myeloid/erythroid ratio		0.95	0.88	1.21∼2.16	1.24∼1.74

^1^Standard deviation. ^*∗*^Reference previously published by Jain [5]. ^*∗∗*^Reference interval established from the values found in the 57 cats without hematological alterations evaluated in this work.

**Table 5:** Quantitative results of the cytological evaluation of bone marrow aspirates from 22 clinically healthy cats with neutrophilia.

Lineage	Evaluated cells (%)	Mean	S.D.^1^	Median	25%	75%	pIR^*∗*^	eIR^*∗∗*^
Myeloid		54	7.1	53.3	48.9	58		
	Myeloblast	1.5	0.8	1.2	0.8	1.9	0∼0.4	0.8∼2.4
	Promyelocyte	2.9	1.3	3	1.9	3.3	0∼3	1.9∼5.3
	Neutrophilic myelocyte	5.1	2.2	5	3.6	6	4.4∼13.2	2.6∼6.4
	Neutrophilic metamyelocyte	3.9	1.2	3.6	3.2	4.8	0.6∼8.0	2.5∼5.3
	Rod neutrophil	11.1	5.6	9.8	8.1	11.6	12.8∼16.6	5.1∼10.5
	Neutrophil	26.1	4.7	27	24.3	28.9	6.8∼22.0	22.2∼30.4
	Eosinophilic myelocyte	0.2	0.3	0	0	0.4	0.8∼3.2 (sum of eosinophilic series)	0∼1
	Eosinophilic metamyelocyte	0.5	0.4	0.4	0.2	0.7		0∼1.2
	Rod eosinophil	0.7	0.6	0.6	0.4	1		0.4∼1.6
	Eosinophil	1.8	1.1	1.6	0.9	2.3		0.8∼3.2
	Basophilic series	0.2	0.2	0	0	0.4	0∼0.4	0∼0.8

Erythroid		35.1	5.7	35.4	32.8	37.3		
	Rubriblast	0.8	0.5	0.8	0.5	1.2	0∼0.8	0.6∼1.6
	Prorubricyte	5.8	2.7	5.6	3.5	7.3	0∼1.6	2.9∼6.9
	Rubricyte	19.8	3	19.6	18.6	21.2	10.2∼29.4	17.7∼25.1
	Metarubricyte	8.7	3.2	9	6.5	10.3	1.0∼10.4	5.9∼10.9

Others		12	3.55	13.2	9.5	14.7		
	Lymphocyte	8.7	2.9	9.1	6.9	10.8	11.6∼21.6	6∼12
	Plasma cell	1.4	0.9	1.2	0.8	1.9	0.2∼1.6	0.1∼2.7
	Monocyte	1.6	0.9	1.6	1.1	2	0∼0.2	0.7∼2.1
	Megakaryocytic series	0.3	0.3	0.4	0	0.4	Rare	0∼0.8

Myeloid/erythroid ratio		1.55	0.31	1.46	1.3	1.74	1.21∼2.16	1.24∼1.74

^1^Standard deviation. ^*∗*^Reference previously published by Jain [5]. ^*∗∗*^Reference interval established from the values found in the 57 cats without hematological alterations evaluated in this work.

**Table 6:** Quantitative results of the cytological evaluation of bone marrow aspirates from 23 clinically healthy cats with eosinophilia and basophilia.

Lineage	Evaluated cells (%)	Mean	S.D.^1^	Median	25%	75%	pIR^*∗*^	eIR^*∗∗*^
Myeloid		53.8	6.3	53.8	51.7	56.8		
	Myeloblast	1.6	0.8	1.4	1	2.2	0∼0.4	0.8∼2.4
	Promyelocyte	3.1	1.4	3.2	1.7	3.8	0∼3	1.9∼5.3
	Neutrophilic myelocyte	5.5	2.7	5.4	3.5	7.4	4.4∼13.2	2.6∼6.4
	Neutrophilic metamyelocyte	4.2	1.2	4	3.3	4.9	0.6∼8.0	2.5∼5.3
	Rod neutrophil	10	4.8	10	7.1	11.1	12.8∼16.6	5.1∼10.5
	Neutrophil	25.3	4.7	25.8	24	27.2	6.8∼22.0	22.2∼30.4
	Eosinophilic myelocyte	0.3	0.4	0.2	0	0.4	0.8∼3.2 (sum of eosinophilic series)	0∼1
	Eosinophilic metamyelocyte	0.5	0.4	0.4	0.4	0.7		0∼1.2
	Rod eosinophil	0.9	0.6	0.8	0.5	1.2		0.4∼1.6
	Eosinophil	2.1	1.4	1.6	1.2	2.4		0.8∼3.2
	Basophilic series	0.3	0.3	0.4	0	0.4	0∼0.4	0∼0.8

Erythroid		35.2	6.1	35	32.8	36.7		
	Rubriblast	1.07	0.7	1	0.7	1.5	0∼0.8	0.6∼1.6
	Prorubricyte	6.8	2.9	5.6	4.3	7.8	0∼1.6	2.9∼6.9
	Rubricyte	20	3.4	20.4	18.8	22.1	10.2∼29.4	17.7∼25.1
	Metarubricyte	7.82	3.3	7.6	5.9	8.7	1.0∼10.4	5.9∼10.9

Others		11.3	3.3	11.8	9.5	13.6		
	Lymphocyte	8	2.6	8	6.7	9.3	11.6∼21.6	6∼12
	Plasma cell	1.4	0.9	1.2	0.8	1.8	0.2∼1.6	0.1∼2.7
	Monocyte	1.6	1	1.8	0.9	2.3	0∼0.2	0.7∼2.1
	Megakaryocytic series	0.3	0.2	0.4	0	0.5	Rare	0∼0.8

Myeloid/erythroid ratio		1.55	0.31	1.38	1.46	1.7	1.21∼2.16	1.24∼1.74

^1^Standard deviation. ^*∗*^Reference previously published by Jain [5]. ^*∗∗*^Reference interval established from the values found in the 57 cats without hematological alterations evaluated in this work.

**Table 7:** Quantitative results of the cytological evaluation of bone marrow aspirates from 8 clinically healthy cats with lymphocytosis.

Lineage	Evaluated cells (%)	Mean	S.D.^1^	Median	25%	75%	pIR^*∗*^	eIR^*∗∗*^
Myeloid		54.5	7.8	52.8	49	50.7		
	Myeloblast	1.5	0.7	1.2	0.9	1.2	0∼0.4	0.8∼2.4
	Promyelocyte	3.1	1.6	3.2	2.6	3.2	0∼3	1.9∼5.3
	Neutrophilic myelocyte	5.3	1.2	5.5	4.5	5.5	4.4∼13.2	2.6∼6.4
	Neutrophilic metamyelocyte	3.8	1.3	3.5	2.9	3.5	0.6∼8.0	2.5∼5.3
	Rod neutrophil	12.4	7.1	11	8.4	11	12.8∼16.6	5.1∼10.5
	Neutrophil	25.9	2.4	25.2	24.6	25.2	6.8∼22.0	22.2∼30.4
	Eosinophilic myelocyte	0	0	0	0	0	0.8∼3.2 (sum of eosinophilic series)	0∼1
	Eosinophilic metamyelocyte	0.3	0.2	0.3	0.2	0.3		0∼1.2
	Rod eosinophil	0.5	0.3	0.4	0.4	0.4		0.4∼1.6
	Eosinophil	1.4	0.6	1.4	0.9	1.4		0.8∼3.2
	Basophilic series	0.1	0.2	0	0	0	0∼0.4	0∼0.8

Erythroid		36.4	2.25	36.4	34.7	36.4		
	Rubriblast	0.9	0.6	0.8	0.5	1.2	0∼0.8	0.6∼1.6
	Prorubricyte	5.7	0.9	6	5.2	6.4	0∼1.6	2.9∼6.9
	Rubricyte	20.6	2.1	20.4	19.4	21.4	10.2∼29.4	17.7∼25.1
	Metarubricyte	7.9	1.9	8.8	7.4	9.2	1.0∼10.4	5.9∼10.9

Others		12	3.8	14.2	9.2	14.7		
	Lymphocyte	10.2	4	11.3	7.4	13	11.6∼21.6	6∼12
	Plasma cell	1.4	0.7	1.4	0.8	1.9	0.2∼1.6	0.1∼2.7
	Monocyte	1.3	0.7	1.2	0.8	1.8	0∼0.2	0.7∼2.1
	Megakaryocytic series	0.3	0.2	0.3	0.1	0.4	Rare	0∼0.8

Myeloid/erythroid ratio		1.42	0.14	1.41	1.34	1.52	1.21∼2.16	1.24∼1.74

^1^Standard deviation. ^*∗*^Reference previously published by Jain [5]. ^*∗∗*^Reference interval established from the values found in the 57 cats without hematological alterations evaluated in this work.

**Table 8:** Quantitative results of the cytological evaluation of bone marrow aspirates from 12 clinically healthy cats with monocytosis.

Lineage	Evaluated cells (%)	Mean	S.D.^1^	Median	25%	75%	pIR^*∗*^	eIR^*∗∗*^
Myeloid		56.3	7.92	54.3	52.9	56.5		
	Myeloblast	1.5	1	1.1	0.8	2.1	0∼0.4	0.8∼2.4
	Promyelocyte	3.1	1.6	3.1	1.6	3.6	0∼3	1.9∼5.3
	Neutrophilic myelocyte	5.7	2.8	5.5	4	6.5	4.4∼13.2	2.6∼6.4
	Neutrophilic metamyelocyte	3.9	1.5	3.8	2.7	4.8	0.6∼8.0	2.5∼5.3
	Rod neutrophil	12.6	7.9	9.7	7.8	13.7	12.8∼16.6	5.1∼10.5
	Neutrophil	25.6	4.4	25.1	24.2	28.2	6.8∼22.0	22.2∼30.4
	Eosinophilic myelocyte	0.4	0.3	0.4	0.1	0.5	0.8∼3.2 (sum of eosinophilic series)	0∼1
	Eosinophilic metamyelocyte	0.6	0.4	0.5	0.4	0.8		0∼1.2
	Rod eosinophil	0.8	0.4	0.8	0.5	1		0.4∼1.6
	Eosinophil	1.7	0.9	1.7	1.1	2		0.8∼3.2
	Basophilic series	0.3	0.3	0.4	0	0.4	0∼0.4	0∼0.8

Erythroid		34.2	5.95	34.8	31.7	37.5		
	Rubriblast	1.1	0.7	1	0.7	1.3	0∼0.8	0.6∼1.6
	Prorubricyte	5.3	2.4	4.9	4	6.7	0∼1.6	2.9∼6.9
	Rubricyte	19.7	3.9	19.6	18.9	22.7	10.2∼29.4	17.7∼25.1
	Metarubricyte	8	2.3	8.4	5.9	9.6	1.0∼10.4	5.9∼10.9

Others		12.5	3.55	12.7	11.9	15.5		
	Lymphocyte	8.8	3	8.8	8.1	10.6	11.6∼21.6	6∼12
	Plasma cell	1.4	0.9	1.2	0.9	1.7	0.2∼1.6	0.1∼2.7
	Monocyte	1.6	1.1	1.5	0.9	1.9	0∼0.2	0.7∼2.1
	Megakaryocytic series	0.4	0.4	0.3	0	0.7	Rare	0∼0.8

Myeloid/erythroid ratio		1.54	0.3	1.5	1.34	1.68	1.21∼2.16	1.24∼1.74

^1^Standard deviation. ^*∗*^Reference previously published by Jain [5]. ^*∗∗*^Reference interval established from the values found in the 57 cats without hematological alterations evaluated in this work.

**Table 9:** Quantitative results of the cytological evaluation of bone marrow aspirates from 17 clinically healthy cats with lymphopenia.

Lineage	Evaluated cells (%)	Mean	S.D.^1^	Median	25%	75%	pIR^*∗*^	eIR^*∗∗*^
Myeloid		51.7	4.26	51.4	48.4	54.8		
	Myeloblast	2	0.7	1.6	1.6	2.4	0∼0.4	0.8∼2.4
	Promyelocyte	2.9	0.7	2.6	2.4	3.4	0∼3	1.9∼5.3
	Neutrophilic myelocyte	5.3	1.6	4.8	4.4	6.2	4.4∼13.2	2.6∼6.4
	Neutrophilic metamyelocyte	3.8	0.9	3.8	3.2	4.6	0.6∼8.0	2.5∼5.3
	Rod neutrophil	7.4	2.5	7	5.8	8.4	12.8∼16.6	5.1∼10.5
	Neutrophil	25.8	3.2	25	24.2	26.6	6.8∼22.0	22.2∼30.4
	Eosinophilic myelocyte	0.5	0.5	0.4	0.2	0.8	0.8∼3.2 (sum of eosinophilic series)	0∼1
	Eosinophilic metamyelocyte	0.5	0.4	0.4	0.2	0.8		0∼1.2
	Rod eosinophil	1	0.4	1	0.8	1.4		0.4∼1.6
	Eosinophil	2.3	1.1	2.4	1.4	3		0.8∼3.2
	Basophilic series	0.2	0.2	0	0	0.4	0∼0.4	0∼0.8

Erythroid		36.1	3.9	34.4	33	38		
	Rubriblast	1.2	0.7	1	0.8	1.4	0∼0.8	0.6∼1.6
	Prorubricyte	5.3	2.4	5.2	3.6	6.8	0∼1.6	2.9∼6.9
	Rubricyte	21.1	3	20.6	19	22.8	10.2∼29.4	17.7∼25.1
	Metarubricyte	8.5	2.1	8.4	7.2	9.6	1.0∼10.4	5.9∼10.9

Others		11.4	3.28	12	9.2	13.6		
	Lymphocyte	8.2	3	8.8	6	9.8	11.6∼21.6	6∼12
	Plasma cell	1.4	0.9	1.2	0.6	2	0.2∼1.6	0.1∼2.7
	Monocyte	1.5	0.8	1.6	0.8	2	0∼0.2	0.7∼2.1
	Megakaryocytic series	0.3	0.2	0.4	0	0.4	Rare	0∼0.8

Myeloid/erythroid ratio		1.46	0.24	1.27	1.5	1.65	1.21∼2.16	1.24∼1.74

^1^Standard deviation. ^*∗*^Reference previously published by Jain [5]. ^*∗∗*^Reference interval established from the values found in the 57 cats without hematological alterations evaluated in this work.

## Data Availability

The epidemiological questionnaires and data transcribed in Excel data used to support the findings of this study are available from the corresponding author upon request.
